# Meaning-Making Process Related to Temporality During Breast Cancer Traumatic Experience: The Clinical Use of Narrative to Promote a New Continuity of Life

**DOI:** 10.5964/ejop.v12i4.1150

**Published:** 2016-11-18

**Authors:** Maria Luisa Martino, Maria Francesca Freda

**Affiliations:** a SInAPSi Centre, University of Naples Federico II, Naples, Italy; b Department of Humanistic Studies, University of Naples Federico II, Naples, Italy; VA Boston Healthcare System (Massachusetts Veterans Epidemiology Research and Information Center) Boston, MA, USA

**Keywords:** breast cancer, meaning-making, health, trauma, temporality, narrative

## Abstract

Previous research has agreed that meaning-making is a key element in the promotion of patients’ well-being during and after a traumatic event such as cancer. In this paper, we focus on an underestimated key element related to the crisis/rupture of this meaning-making process with respect to the time perspective. We consider 40 narratives of breast cancer patients at different times of treatment, undergoing chemotherapy and biological therapy. We collected data through writing technique. We performed an interpretative thematic analysis of the data and highlighted specific ways to signify time during the different treatment phases. Our central aspect “the time of illness, the illness of time” demonstrates that the time consumed by illness has the risk of becoming an illness of time, which transcends the end of the illness and absorbs a patient’s past, present, and future, thus saturating all space for thought and meaning. The study suggests that narrative can become a therapeutic and preventive tool for women with breast cancer in a crisis of temporality, and enable the promotion of new semiotic connections and a specific functional resynchronization with the continuity/discontinuity of life. This is useful during the illness and medical treatment and also after the treatment.

Researchers have generally found that breast cancer is a traumatic experience that disrupts women’s lives, particularly with regard to sense of being female, body, fertility, and motherhood (Cordova et al., 2007; Kirkman et al., 2014; Koutrouli, Anagnostopoulos, & Potamianos, 2012; Liamputtong & Suwankhong, 2015; Mehnert & Koch, 2007). Most studies have evaluated the negative outcomes of trauma or the effect of returning to normal life and work after a traumatic experience (Calhoun & Tedeschi, 2014; Gotay, 2010; Johnsson, Fornander, Rutqvist, & Olsson, 2010; Lindstrom & Triplett, 2010). However, qualitative research on the meaning-making of the traumatic experience, especially focusing on subjective temporality during the different breast cancer treatment phases, is limited. Researchers have been interested in the concept of time perspective since 1930, and different theoretical perspectives regarding the perspective have evolved (Bakhtin, 2002; Boniwell & Zimbardo, 2003; Brockmeier, 2000; Frank, 1998; Hoornaert, 1973; Minkowski, 1933; Ricoeur, 1980). Life-threatening illnesses enforce a quest for meaning, which must be addressed (Horowitz, 1986; Janoff-Bulman, 1992; Neimeyer, 2006). It represents a temporal framework crisis that becomes an important activator in the construction and transformation of meaning (Del Vecchio Good, Munakata, Kobayashi, Mattingly, & Good, 1994; Gillies & Neimeyer, 2006; Hall, 2011; Martino, Onorato, & Freda, 2015; Pals & McAdams, 2004; Waters, Shallcross, & Fivush, 2013). Women with breast cancer experience changes in their body image, the sense of being female, and sexuality, and the trauma interrupts their meaning-making processes, thus building the organization of the time perspective and subjective experiences (Martino, Onorato, D’Oriano, & Freda, 2013). Furthermore, it leads to changes in their representation of the past and plans for the future (Fobair et al., 2006; Koopman, Hermanson, Diamond, Angell, & Spiegel, 1998; Lund-Nielsen, Müller, & Adamsen, 2005; Pelusi, 2006; Sadler-Gerhardt, Reynolds, Britton, & Kruse, 2010).

From a clinical perspective, supporting the meaning-making process with respect to a traumatic experience is an essential foundation for adapting, integrating trauma, and developing patient’s well-being (Davis, Wortman, Lehman, & Silver, 2000). It is crucial because it appears to be linked to health outcomes and reduction of symptoms (Bower, Kemeny, Taylor, & Fahey, 2003; Park, Edmondson, Fenster, & Blank, 2008; Tolstikova, Fleming, & Chartier, 2005). This promotes cognitive and emotional processing, thus integrating the event into the patient’s personal life story (Horowitz, 1986; Janoff-Bulman, 1992). Meaning-making has positive effects, including protection against anxiety and reduced anxiety (Davis et al., 2000). Life with little or no meaning causes existential distress, and recent research has demonstrated that meaning-making improves breast cancer patients’ self-esteem, optimism, and self-efficacy (Freda et al., 2016; Lee, Cohen, Edgar, Laizner, & Gagnon, 2006). Individuals who successfully complete the process of searching for meaning often emerge with a sense of renewal, greater self-awareness, and personal growth, as well as a greater appreciation for life, nature, and compassion for others (Lee, 2008).

## Breast Cancer, Narrative, and Temporality

Studies focused on the illness narratives of women with breast cancer highlighted how the onset of cancer is narrated as a biographical disruption, temporal fracture, and discontinuity affecting temporal frameworks (Bülow & Hydén, 2003; Freda, Dicé, Auricchio, Salerno, & Valerio, 2015; Hillmann, 1984; Kleinman, 1988). Temporality is a basic and constituent dimension of human existence; everything is developed and signified in a time frame (Barak & Leichtentritt, 2014; Brockmeier, 2000; Broom & Tovey, 2008; Carr, Teucher, & Casson, 2014). A complex interplay of dynamic processes with multilayered meanings (biological, psychological, subjective, cultural, and social) guides an individual’s perceptual-cognitive phenomena, actions, social relationships, and life choices (Sato & Valsiner, 2010; Tronick, 2009).

Generally, narratives of breast cancer generate a meaning fracture in patients, implying a representation of time that is characterized by a sense of uncertainty about the future, avoidance, and a sense of interruption of one’s lifetime that is strong enough to continue over the years (Arena et al., 2007; De Luca Picione, Dicè, & Freda, 2015; Farley, Minkoff, & Barkan, 2001; Hjelmblink & Holmström, 2006; Martino & Freda, 2016; Rasmussen & Elverdam, 2007). Disrupted time organization caused by the traumatic experience immobilizes the mind in an infinite present, sometimes characterized by an obsession with the nostalgic past and sometimes with the future (Bonomi, 2003). The dramatic discontinuity, a result of the quality of the event, opens a dichotomous view of life; staying alive in a past full of expectations and projects or staying rooted in the certainty of a future in which anything is possible. The crisis of temporality can lead to a block in the present, with no scope for progress (Foa, 1997; Minkowski, 1933).

Within a constructivist and semiotic perspective (Efran, McNamee, Warren, & Raskin, 2014; Valsiner, 2014), the narration of illness promotes the possibility of recounting past events and emotions through memory. Narration calls to mind events, in the *hic et nunc*, supporting a meaning-making process and semiotic transformation of the experience. The narration, in its different devices (e.g., Esposito, Freda, & Bosco, 2015; Margherita, Gargiulo, & Martino, 2015), allows access to past events through memory and brings them to the present. The individual can comprehend these events, reconstruct the meaning of his/her own life story, and gradually construct him/herself within a specific cultural and temporal perspective (Angus & McLeod, 2004; Bruner, 1992; Frank, 1998; Hermans, 2003; Neimeyer, 2002, 2004, 2006; Pals & McAdams, 2004; Thomas-MacLean, 2004).

Temporality refers to the way a semiotic net is constructed. Therefore, a narrative told in the present is a semiotic construction and reconstruction of past experiences and the context in which it is narrated (Brockmeier, 2000). The narrative expresses everything about temporality; it is seen as a model of time and a linguistic and psychological framework that arranges the diachronic dimension of our activities. It becomes the most suitable form for constructing and reconstructing time, and it expresses an oscillation between the past and present while also sometimes referring to the future (Brockmeier, 2000).

Narrative and temporality are strongly correlated. Time becomes important for organizing the narrative experience. Therefore, the narrative of traumatic experiences assumes the function of reconstructing the story of the continuity of life, including the personal story context, consolidating the interruption of time and creating a new connection between the continuity and discontinuity of the experience. This constructs a meaning that bridges the past, present, and future (Freitag, Grimm, & Schmidt, 2011; Hydén, 1997; Murray, 2007).

To increase awareness of the meaning-making of temporality during breast cancer, we explored the themes according to which women organize the meanings of their written narratives about being illness time. In particular, we extensively studied the relationship between the different phases of chemotherapy and biological therapy phases (Martino, Onorato, D’Oriano, & Freda, 2013) and potential different ways to signify the time of illness. This highlighted the clinical role and function of the narrative in a crisis of temporality. When discussing the two phases of the illness, we refer to not only the two different aspects of treatment but also the two different moments of the traumatic experience, that is, the phase of chemotherapy, which is closest to the traumatic experience of being diagnosed with the disease and its subsequent uncertainty, and the phase of biological therapy, which is closer to the phase of life in which reconstruction begins and patients begin to return to everyday life experiences.

## Methods

### Participants and Tools

Participants included patients at two hospitals for the care and assistance of oncological illness in Southern Italy during 2014. The research was approved by the ethical committees of the participating hospitals. We considered 45 women with non-metastatic breast cancer undergoing drug treatment after surgery. Participants’ cancer stage, phase of treatment, and year of diagnosis were determined with help from medical records and medical staff. Participation in the research intervention was voluntary, and informed consent was obtained from all participants. We invited patients to participate in the study by asking them to come for a *vis-a-vis* appointment. Five women declined because of lack of interest or time. Thus, the final study group comprised women with non metastatic breast cancer: 20 women in the first cycle of chemotherapy diagnosed for 6 months (*M*_Age_ = 45.3, *SD* = 10.6) and 20 women in biological therapy diagnosed for 12 months (*M*_Age_ = 49.1, *SD* = 8.78).

Within the paradigm of Expressive Writing Technique (Pennebaker, 2002), we constructed three different writing tasks according to the framework of the Guided Written Disclosure Protocol (Duncan et al., 2007; Gidron et al., 2002). In every meeting, the participants dealt with a different traumatic aspect of breast cancer: factual aspects, thoughts and feelings, change, and future perspective. This guided the participant to a narrative reconstruction of their traumatic experience. In the first session, the participants were asked to write events as they occurred and evolved eventually, in chronological order. In the second session, they were invited to write about their emotions at these times. In the last session, they were asked to think about their future, projects for the future, the effects that the disease had on them, and how they would deal with such difficult experiences in the future. The writing sessions were conducted approximately every 10–15 days to promote a transformation process with respect to their experiences between meetings. Every meeting lasted 30–40 min.

### Data Analysis

Within a socio-constructivist and semiotic framework (Efran, McNamee, Warren, & Raskin, 2014; Valsiner, 2014), we analyzed the collected narratives using interpretative thematic analysis of the written text according to the constructivist grounded theory approach (Charmaz, 2003, 2006). Contrary to the classic grounded theory approach (Glaser & Strauss, 1967), this analysis is suitable for highlighting the constructivist focus on the interpreted understandings of the participants’ meaning. The ATLAS.ti.7. software, Computer Assisted Qualitative Data Analysis software, and a qualitative data analysis program were used to explore, interconnect, and analyze all data sources in depth. The analysis enabled the creation of thematic categories, which explain the interpretation of the meaning of these categories. Using progressive abstraction, the categories were homogenized to reduce redundancies and subsequently aggregated within four families connected by a core category as per an interpretation criterion.

## Results

**Figure 1:**
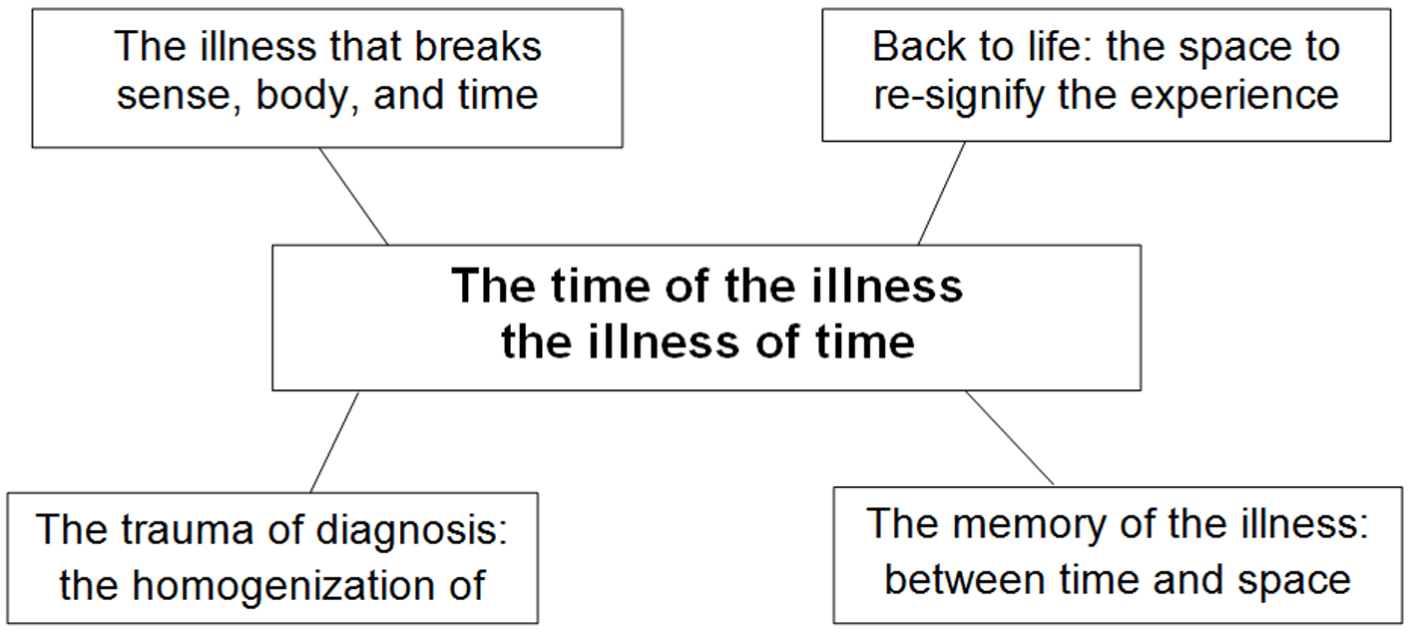
The time of illness, the illness of time.

Our findings are summarized in Figure 1, which indicates the results from the core category and the four families that emerged from the analysis.

Our findings were organized into four categories that combine the narratives of women in the chemotherapy and biological therapy phases within different classes. In the upper left quadrant is the “the illness that breaks sense, body, and time” category; in the lower left quadrant is the “the trauma of diagnosis: the homogenization of time” category; in the upper right quadrant is the “back to life: the space to resignify the experience” category; and in the lower right quadrant is the “the memory of the illness: between time and space” category.

The core category, “the time of illness, the illness of time” is a semiotic connector of the different worlds and ways of living through the time of illness. The narratives were collected into Italian language and the authors translated the narrative extracts from Italian into English language.

### The Illness That Breaks Sense, Body, and Time and the Trauma of Diagnosis

This category comprises themes regarding the “time/not time” of diagnosis. Uncertainty and the precariousness of meaning-making were predominant in the narratives of women undergoing chemotherapy (Hjelmblink & Holmström, 2006; Rasmussen & Elverdam, 2007). The uncertainty and precariousness caused by the diagnosis are revoked in the narration’s *hic et nunc*, that is, the difficulty of constructing a meaning regarding what has happened and what will happen: “It was as if my head was elsewhere, as if I were muffled; the brain sensed what was happening but at the same time, I wondered if it was possible.” The narrative expressed and signified the experience of diagnosis as an internal watershed time that splits the emotion and thought regarding the traumatic communication of diagnosis like a liminality time (Blows, Bird, Seymour, & Cox, 2012; Gargiulo et al., 2014).

Thinking lead to acting following routine care, clinical examinations, and searching for a solution. However, following the diagnosis, the patients began to seek an explanation for what had happened: “These feelings and confusion, like a vortex, lasted about a week; then suddenly there was a bright light, the doors were opened, and thoughts were changing because events were changing.” The diagnosis is immersed in a time full of other catastrophic events, often similar in nature. In the narrative space, the breast cancer diagnosis becomes negative and has a salient effect on everything: “Earlier, almost a sign of fate, my father had died of cancer; I looked at myself in the mirror. I see the same face that my father had before he died.” The diagnosis breaks all of life’s certainties and leads to the unknown: “As soon as they gave me the diagnosis, I fell upon the world; I thought it was not real. My head was bursting with thoughts, and I thought about what would happen … without results.” Time appears to be blocked in that particular instant, which consumes the construction of meaning.

For women undergoing biological therapy, we found a different and specific narrative of the onset of breast cancer. These women described a lack of meaning caused on hearing the diagnosis. However, these women could reevaluate the time of diagnosis and interpret it in the *hic et nunc* as eliminating uncertainty; thus, they pointed the way forward: “Fortunately, the diagnosis arrived; it arrived on time. I called my husband, and I communicated to him that the next day I would have to undergo the surgical operation. I was speechless.” Women in the biological therapy phase describe that the communication of diagnosis produces a process of meaning-making where anger relents to the resumption of life and opens a space for reflection. Thus, the diagnosis can be seen as a “salvation” that enables the dissolution of doubts and uncertainties, indicating a future direction.

In these narratives, the women describe their experiences of condensation as a defense against the feeling of time slipping away. In the traumatic moment of the diagnosis, time appears to be too short and too close to the end: “It was Thursday, Thursday afternoon. I was asked to undergo the surgical operation immediately. Now, not next Friday? No, too late, better the next day.” The illness is not a chaotic category and is inscribed into her personal story. In some cases, it was seen as being inevitable, a part of life: “A kind of time bomb, which inevitably would break out sooner or later … Maybe it had to happen.”

The concrete memory of the onset of the illness is formed differently in the two groups of women. For women in the chemotherapy phase, the time of the communication of the diagnosis is when time stopped and this remains a concrete and indelible space in their memory. For women in the biological therapy phase, the memory of the onset of the illness leads to a future-oriented temporality.

### The Space to Resignify the Experience and the Memory of the Illness

This category condensed the question of space and future projects. The future, an undefinable time because it is unknown, is of particular significance in the illness narratives. The patients describe the future as flattened in the present, an eternal present. The past is a distant memory and the future is unthinkable. This lack of perspective is particularly evident in the narratives of women undergoing chemotherapy. The future is now and no perspective can go beyond the present, as they wrote: “It is a period of stalemate; I do not make plans because there is a risk that I cannot see them through. I try to live day-by-day; I cannot see anything in front of me, neither positive nor negative.”

Life appears static in these narratives. Life is not seen as a process with its own trajectory and direction toward a definite goal. Thus, patients live in a state of paralyzed memory, overwhelmed by an experience that is absorbing all possible developments like a black hole (Brockmeier, 2000). It is as if an illness of the body becomes an illness of time; the body is “blocked” after the diagnosis and treatment, therapies and time separate it from its natural flow, and it becomes a passive body, which is resigned to submit to medical treatment; time is left to drain passively, without the patient actually living it. Passivity toward the flow of time creates a sense of resignation and a sense of surrendering to fate, as expressed by the patients: “What will be, will be.” Therefore, the path of the illness passively alters over time, floating on the flow of events: “I did not have the enthusiasm to do things because everything seemed useless. I went ahead by inertia; I was a passive viewer of my life.” This expresses a lack of the vital force that creates the future.

The narratives of women undergoing biological therapy emphasize on the future. The future is not blocked, rather it starts to be symbolized and viewed. Biological therapy occurs at a later phase when patients begin to think about short-term projects but are always uncertain about long-term plans: “I look to the future with caution. I try to enjoy the little things. I cannot make long-term plans. My thoughts about the future do not go far.” Here, temporality carefully begins to dive back into the stream that had been stolen earlier. Meaning can be attributed to the traumatic event, considering the unusual circumstances as reference, and the patient can slowly resume the other trajectories of life, such as family, children, and friends (Ashing-Giwa, Padilla, Bohórquez, Tejero, & Garcia, 2006). The illness becomes a part of the memory; the past and the future exist only in relation to the present. This event could not be born from that past and, therefore, must be seen as something that leads to the future (Minkowski, 1933). Therefore, during biological therapy, the women signify the chemotherapy period as a parenthesis, a time suspended and removed from the natural flow of time: “I lived the dark period of chemotherapy for more than a month, a month removed from life; you are dead, not alive.” The clear separation between the patient’s life before and after the diagnosis is the most prominent; it represents a suspended period of life in expectation of healing or death.

### The Time of Illness, the Illness of Time

Our core category represents a semiotic connector of the specific and different ways of organizing the narration of breast cancer. When the crisis caused by the illness is not signified, (for example, women undergoing chemotherapy), the temporal perspective cannot be expressed. Thus, the patient cannot develop in a manner that enables the implementation of relational systems. The illness acquires the configuration of a real illness of time, represented by the loss of continuity of life before the crisis and the loss of the ability to integrate and connect experiences.

When the illness replaces an experience integrated into a broader framework of life, we observe that, in spite of the existence of painful memories and loss, the illness of time starts to become the time of illness, a period of semiotic transition that requires resignifying their lifestyles, reconstructing their the pasts, reconsidering their relationships, and reviewing their futures. The illness does not signify a crystallized meaning of one’s entire life but a process of transformation that offers the opportunity to construct and shape new connections within the trajectories of the continuity and discontinuity of life. Therefore, all women configure the illness and its time as an event of discontinuity that undermines the continuity systems and puts a strain on the mediation processes between continuity and discontinuity, thus consuming the construction of meaning.

## Conclusions and Clinical Implications

Our study is limited by the small sample size and its qualitative nature. However, our findings showed that time assumes particular meanings during different treatment phases of breast cancer. We also observed efforts to construct mediation processes of the relationship between continuity and discontinuity of life experiences. Within a diachronic framework, the narration becomes a therapeutic and preventive device that promotes the integration and synchronization of the traumatic experience in relation to the continuity and discontinuity of social life. Narratives are used to objectify and distance oneself from one’s problems to better understand, establish meaning, develop greater self-knowledge, and decrease emotional distress. In this study, we highlighted that the narrative changes over time. It is a synchronic process of experience reorganization and a diachronic process that continuously evolves and is organized in time (De Luca Picione & Freda, 2016).

These new semiotic connections do not imply that they are the best in terms of integration; however, the formation of these connections is a resynchronization process with the illness and with the continuity and discontinuity of life where different configurations of time can coexist (Barak & Leichtentritt, 2014). The narration of trauma associated with breast cancer indicates a temporal perspective crisis and is semiotically significant in the search for connections aimed at constructing a time of illness and reducing the illness of time. Toward the end of treatment, we believe that the time of illness is at risk of becoming an illness of time, absorbing the past, present, and future and consuming all thoughts and meaning. We suggest that clinical health psychologists and health operators examine the importance of the personal narratives of women with breast cancer (Zannini, Cattaneo, Jankovic, & Masera, 2014). This represents a process of psychosocial recovery of a shared temporality in which the patient may live in her past and future and reconnect with psychosocial actors (family, friends, and children).

The narrative device, in its various forms, assumes the therapeutic and preventive function of a semiotic device in relation to continuity and discontinuity of life experiences when seeking a new temporal synchrony based on the importance of meaning-oriented transformation (Tang et al., 2007).

Thus, we assume that continuing with life is based on the possibility of discussing the relationship between the continuity and discontinuity of patients’ experiences (Sato & Valsiner, 2010). The different phases of breast cancer treatment have personal meaning, and the narrative enables the communication of this meaning. Through narration, we can meet the challenges of life in numerous ways. In conclusion, we suggest that narration promotes a dynamic resynchronization with the illness, creating a dynamic balance (Boniwell, Osin, Linley, & Ivanchenko, 2010). This respects the different treatment phases, both at the termination of the illness and when preventing the time of illness from becoming an illness of time from the time of diagnosis to the conclusion of treatment (De Feudis, Lanciano, & Rinaldi, 2015). For future research, we suggest integrating qualitative analysis with external indicators to assess the effects of the meaning-making process on health during the cancer experience.
